# Effectiveness of the Fixtoe Device® in plantar pressure reduction: a preliminary study

**DOI:** 10.1186/s12891-022-05415-5

**Published:** 2022-05-19

**Authors:** Maria Ruiz-Ramos, Ángel Manuel Orejana-García, Ignacio Vives-Merino, Carmen Bravo-Llatas, José Luis Lázaro-Martínez, Raúl Juan Molines-Barroso

**Affiliations:** 1 Facultad de Enfermería, Fisoterapia y Podología, Clínica Universitaria de Podología, Universidad Complutense de Madrid, Instituto de Investigación Sanitaria del Hospital Clínico San Carlos (IdISSC), Edificio Facultad de Medicina. Pabellón 1. Avda. Complutense s/n, 28040 Madrid, Spain; 2 Área de Gobierno de Tecnologías de la Información y Apoyo Técnico al Usuario – Investigación, Universidad Complutense de Madrid, Madrid, Spain

**Keywords:** Metatarsophalangeal joint, Joint subluxation, Foot, Metatarsalgia, Conservative treatment, Cross-sectional studies

## Abstract

**Background:**

Metatarsalgia is a common foot condition. The metatarsophalangeal stabilizing taping technique described by Yu et al. has shown good clinical results as a provisional treatment in propulsive metatarsalgia. ^35^ The Fixtoe Device®, a novel orthopedic device, intends to simulate stabilizing tape. However, to date, there is no evidence of its effectiveness. The aim of this study was to assess plantar pressure changes using the Fixtoe Device®, in comparison with the traditional method (stabilizing tape) in a young, healthy sample thorough a cross-sectional study.

**Methods:**

Maximal pressure (Kpa) and pressure–time integral (Kpa/s) in the second metatarsal head were measured in twenty-four healthy volunteers. Registers were taken in four different conditions: barefoot, traditional stabilizing tape, Fixtoe Device® without metatarsal pad, and Fixtoe Device® with metatarsal pad.

**Results:**

Mean second metatarsal head maximal pressure and mean pressure–time integral showed statistical difference among the four analyzed conditions (*p* < 0.0001 in both cases). The improvement in maximal pressure and pressure–time integral obtained in each intervention also showed significance (*p* < 0.0001 in both cases). Comparing the improvement of the Fixtoe Device® with and without metatarsal pad with that of tape condition showed a moderate to high and moderate effect size for both peak pressure and pressure–time integral reduction.

**Conclusions:**

The Fixtoe Device® reduces median maximal pressure and median pressure–time integral under the second metatarsal head in healthy young individuals. The Fixtoe Device® shows higher effectiveness than the traditional second metatarsophalangeal joint stabilizing taping technique. To our knowledge, this is the first investigation proving the effectiveness of the recently developed Fixtoe Device® in terms of plantar pressure modification, which leads the way to its use in clinics.

## Background

Metatarsalgia is a common foot condition, although there is no robust statistical data on its prevalence. Previous studies have reported that forefoot pain is relatively more prevalent in feminine middle-aged and elderly patients (rates vary from 19 to 35%). Metatarsalgia is related to the origin of pain in other locations on the foot, and lower limbs and higher fall risk, having a negative impact on the patients’ quality of life. Nevertheless, epidemiological studies do not differentiate whether the pain is related to the metatarsophalangeal joint itself or to other anatomical structures in the forefoot [6, 9, 11, 33].

Propulsive metatarsalgia occurs during the push-off phase of the gait cycle. During this phase, ground reaction forces increase in the forefoot’s region. Metatarsal bones are then exposed to axial compressive forces. The maximal dorsiflexion range in metatarsophalangeal joints is then detected. As a result, the metatarsophalangeal joint capsule and plantar plate are under both tensile and compressive forces.

Overload in certain metatarsophalangeal joints, mainly the second and third, leads to pathologic conditions. Second-space syndrome (also known as pre-dislocation syndrome), causing propulsive metatarsalgia, is characterized by metatarsophalangeal joint instability leading to synovitis and deformity in sagittal and transverse planes as a consequence of joint capsule distension and damage to collateral ligaments and plantar plate [3, 6, 14, 25].

Repetitive overloading under the metatarsal heads causes metatarsalgia. The origin of metatarsal pain is related to the magnitude of the pressure received and the duration of the load [28]. The excess pressure in the area can be reduced by adequately applying conservative or surgical treatment. According to the available literature, plantar pressure reduction in central metatarsal heads is related to pain reduction. In clinical practice, routine plantar pressure measurements are useful to monitor plantar pressure variations before and after treatment [1, 2, 6, 8, 22, 25].

Conservative treatment aims to control biomechanical disorders causing metatarsal overload in order to reduce local plantar pressure and lead the patient to a subclinical condition, slowing down deformity progression [6, 20, 34]. Multiple conservative options have been described, such as nonsteroidal anti-inflammatory drug administration, physical therapy, plantar foot orthosis, cushioning metatarsal pads and footwear modifications [18, 32, 35].

**Fig. 1:**
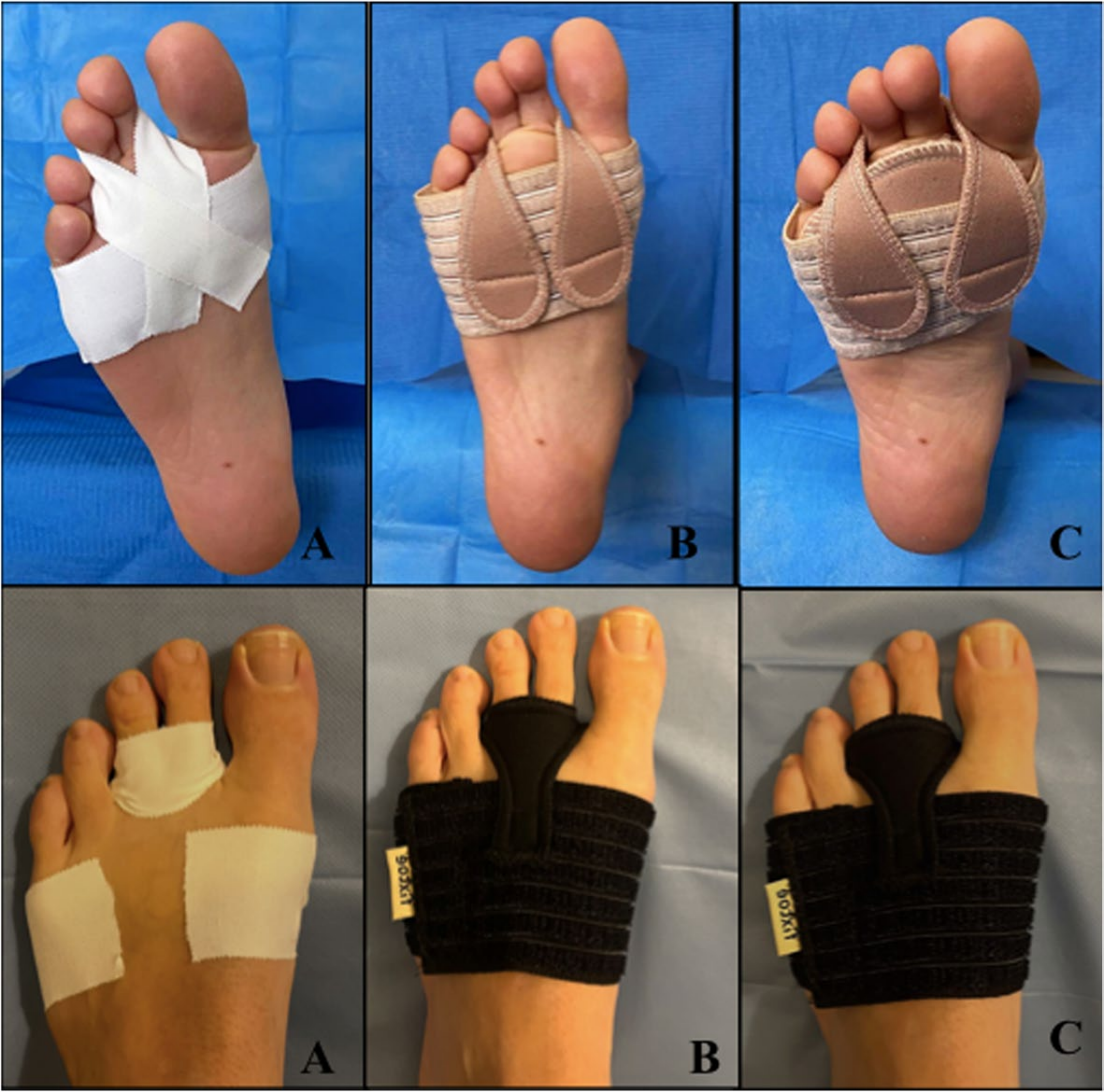
Measured interventions. **A** Traditional stabilizing tape; **B** Fixtoe Device® without metatarsal pad; **C** Fixtoe Device® with metatarsal pad

The metatarsophalangeal stabilizing taping technique described by Yu et al. has shown good results as a provisional treatment in propulsive metatarsalgia, reducing the risk of phalanx dorsal luxation and synovitis, although, in order to be effective, it may have to be carried out for several months [18, 30, 35]. As described, the stabilization technique is made using thin strips of tape (approximately 25 mm wide). The tape is placed dorsally proximal on the toes and fixed in the plantar aspect of the MTP joints, as shown in Fig. 1 [35]. Its main disadvantages are the need to place it at home by the patient and skin lesions by contact with the adhesive [3].

The Fixtoe Device® (Fixtoe Device SL, Elda, Spain), a novel orthopedic device recently designed by Spanish podiatrists, intends to simulate stabilizing tape. It consists of movable elastic straps anchored to an adjustable elastic band, which is placed around the forefoot and a removable metatarsal pad. The straps are placed on the dorsal surface of the forefoot and taken to its plantar aspect by the second, third, or fourth interdigital space, providing a plantarflexion moment to the metatarsophalangeal joints involved (Fig. 1). The straps’ plantar anchoring has a horseshoe discharge function.

The application technique is similar when using traditional stabilizing taping or the Fixtoe Device®. Besides the cushioning pad, the major differences are the greater thickness of the device’s straps (about 2 mm in Fixtoe Device®) and their elasticity.

To date, we are unaware of any investigation that has evaluated the effectiveness of Fixtoe Device® to reduce the plantar pressure. Due to similar characteristics with the taping technique, we hypothesized that the Fixtoe Device® could obtain comparable effects in the reduction of metatarsal plantar pressure [3, 6, 18, 30, 35].

The aim of the study is to assess plantar pressure changes within a comparison between a novel orthopedic device (Fixtoe Device®) and the traditional stabilizing tape [35] in young, healthy individuals.

## Methods

A cross-sectional study and carried out in a podiatry clinic in Madrid, Spain. The design of the study took place during January 2020. Subsequently, 24 individuals were clinically evaluated consecutively and asked to participate. Data records took place from February to March 2020. Participants’ verbal informed consent was obtained, and their rights were protected according to the study protocol approved by the corresponding Ethics Committee.

### Participants

Participants were healthy volunteers ≥ 18 years old who did not refer to pain in the metatarsal region within the last year and did not show lower limb morphological or functional alterations. Individuals with a history of foot and ankle surgery were excluded.

### Variables

#### Clinical evaluation

Clinical measures evaluated in the present investigation were: functional *hallux limitus*, classified as present or absent according to functional *hallux limitus*test positive (present) or negative (absent) results [4]; active extension range of mobility of the first metatarsophalangeal joint, measured with a manual goniometer whose center was placed medial to the center of the first metatarsal head, with one of its arms perpendicular to the floor and the other aligned to the proximal phalanx of the hallux; with the participant standing on its Fick’s angle (expressed in degrees) [16]; and the spatial orientation of the subtalar joint axis, classified as medial, neutral, or lateral according to the technique described by Kirby [21].

All clinical measures were registered by the same investigator.

#### Main outcomes

The main outcome measures were maximal pressure (Kpa) and pressure–time integral (Kpa/s) in the second metatarsal head, measured in each condition.

Maximal pressure and pressure–time integral variations are frequently chosen in the literature as the main outcome measures when evaluating orthopedic devices; since the increase of pressure at a certain area and the duration of the application of the load have previously been related to the origin of pain in the foot [22, 28, 29].

Maximal pressure and pressure–time integral improvement were considered as the decrease between basal condition and each intervention and was expressed as the change in the means (∆).

A 2-m long dynamic pressure measurement system (Footscan® system, RSscan International, 3583 Olen, Belgium) was used to record the main outcome measures. The employed hardware consisted of a 2-m plate with four sensors per cm^2^ and a 3D-Box interface synchronized with a motion capture system. Data were recorded at a 500 Hz measurement frequency and processed using Scientific Footscan® software (RSscan International, 3583 Olen, Belgium).

The plantar pressure register was taken in four different conditions. On the first place, the participants’ basal condition was registered: 1) barefoot. Then, the registers of the three conditions considered as interventions were taken: 2) traditional stabilizing tape, 3) Fixtoe Device® without a metatarsal pad, and 4) Fixtoe Device® with a metatarsal pad.

Second and third metatarsophalangeal joints were stabilized using the taping technique described by Yu et al. in the second condition and holding them with the Fixtoe Device® straps in conditions 3) and 4) (Fig. 1) [35].

A clinician with more than 3 years of experience in the use of the tape technique performed all applications of the different interventions in the dominant foot of each patient.

Following the data collection protocol on our group [24], participants were asked to walk in all the conditions for 3 min in the lab in order to normalize their gait pattern and walked a 1.5-m straight distance before reaching the platform, then the second step on the platform was recorded. This procedure was repeated three times in all conditions.

Another investigator, who was blinded to the order of the application of conditions, performed the plantar pressure register.

### Statistical analysis

Statistical analysis was performed using SPSS statistics version 25.0 for Mac OS (SPSS, Chicago, IL, USA).

The statistical analysis of the results was performed using the mean value of the three registers, calculated for each condition.

Quantitative variables were presented as mean and standard deviation or as median and interquartile range. Qualitative variables were presented as frequencies and percentages. The normal distribution of quantitative variables was tested using the Shapiro–Wilk test.

Analysis of variance for repeated measures was used to explore the difference in maximal pressure and pressure–time integral among the four studied conditions (barefoot vs. stabilizing tape, barefoot vs. Fixtoe Device® without a metatarsal pad, barefoot vs. Fixtoe Device® with metatarsal pad, stabilizing tape vs. Fixtoe Device® without a metatarsal pad, stabilizing tape vs. Fixtoe Device® with metatarsal pad and Fixtoe Device® without metatarsal pad vs. Fixtoe Device® with metatarsal pad), and the change in the means in each intervention. Paired comparisons of the improvement among the three interventions were performed. Bonferroni correction was applied to the paired comparison’s *p*-values and mean differences’ confidence intervals.

To determine the clinical relevance of maximal pressure and pressure–time integral values’ improvement after interventions, the effect size was calculated for each of them with 95% confidence intervals. Cohen’s d was calculated as described by Lenhard and Lenhard for repeated measures with the pooled standard deviation and considering Pearson correlation [23]. Effect size cut-off values were established according to Ferguson’s criteria suggesting a small, moderate, or large, meaningful difference when d equals or exceeds 0.41, 1.15 or 2.7, respectively [7]. *P* values < 0.05 were considered statistically significant, with 95% confidence intervals.

## Results

Data of demographics and clinical evaluation of the 24 healthy individuals included in the study are shown in Table 1. The Shapiro–Wilk test showed a normal distribution for the quantitative variables.

**Table 1:** Participants’ demographics and clinical evaluation

***(n* = *24 participants)***	
*Male n (%)*	11 (45.8)
*Female n (%)*	13 (54.2)
*Age (years). median (IQR)*	24 (23—25)
*BMI (kg/cm*^*2*^*). median (IQR)*	21.95 (19.89–23.83)
*1st MTPJ extensión (degrees). median (IQR)*	46.0 (40–52)
*Functional Hallux Limitus (positive FHL test). n (%)*	8 (33.3)
*Spatial orientation of the STJ axis*	
*Medial STJ axis. n (%)*	19 (79.2)
*Neutral STJ axis. n (%)*	4 (16.7)
*Lateral STJ axis. n (%)*	1 (4.2)

*IQR* Interquartile range, *BMI* Body mass index (kg/cm^2^), *MTPJ* Metatarsophalangeal joint, *FHL test* functional *hallux limitus* test, *STJ* subtalar joint

Table 2 shows the mean maximal pressure and pressure–time integral in the second metatarsal head for the barefoot, stabilizing tape, Fixtoe Device® without metatarsal pad and Fixtoe Device® with metatarsal pad conditions.

**Table 2:** Main outcome measures’ results

	***Pmax (Kpa)***	*∆*	*95% CI*^*a*^	*p-value*^*b*^	***P–T (Kpa/s)***	*∆*	*95% CI*^*a*^	*p-value*^*b*^
*Barefoot*	198.0 (± 13.4)				36.5 (± 2.6)			
*Stabilizing tape*	166.2	31.7	14.9 – 48.5	< .0001	30.2	6.3	2.8 – 9.7	< .001
	(± 12.5)	(± 6.5)			(± 1.9)	(± 1.3)		
*Fixtoe Device®*	125.1	72.9	52.7 – 93.1	< .0001	21.4	15.1	10.4– 19.8	< .0001
	(± 9.7)	(± 7.8)			(± 1.5)	(± 1.8)		
*Fixtoe Device® w/metatarsal pad*	106.2	91.7	67.5 – 116.0	< .0001	18.2	18.2	13.3 – 23.2	< .0001
	(± 9.0)	(± 9.4)			(± 1.5)	(± 1.9)		

*Pmax* maximal pressure, *95% CI* 95% confidence interval, *P/T* Pressure–time integral. Mean (± SEM)

^a^^−^^b^*p*-value and 95% CI refer to the Bonferroni correction showing the improvement (∆) between each intervention and the basal barefoot condition

Plantar pressure decreased with Fixtoe Device® in comparison with the barefoot condition (198.0 ± 13.4 Kpa) in both cases: without metatarsal pad (125.1 ± 9.7 Kpa, *p* < 0.0001 [range, 52.7–93.1]) and with metatarsal pad (106.2 ± 9.0 Kpa; *p* < 0.0001 [range, 67.5–116.0]).

It also showed significance regarding the improvement (∆) in maximal pressure and pressure–time integral obtained in each intervention: ∆ 72.9 (± 7.8) for maximal pressure comparing Fixtoe Device® without metatarsal pad vs. barefoot condition and ∆91.7 (± 9.4) for maximal pressure comparing Fixtoe Device® with metatarsal pad vs. barefoot condition. A posteriori paired-comparison tests with Bonferroni correction showed significance in the improvement in terms of maximal pressure and pressure–time integral with the three studied interventions.

**Fig. 2:**
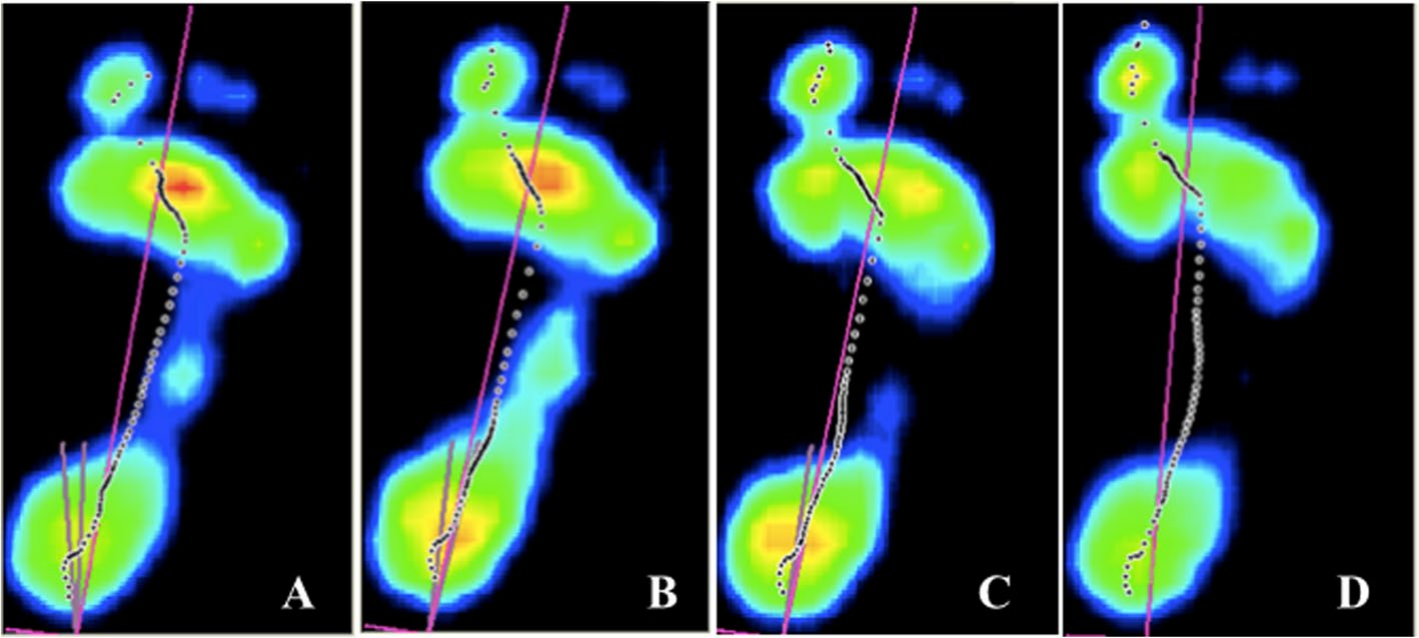
Plantar pressure distribution. **A** Barefoot; **B**. Traditional stabilizing tape; **C **Fixtoe Device® without metatarsal pad; **D** Fixtoe Device® with metatarsal pad

Figure 2 serves as a clear visual example of the variation in pressure distribution in the whole plantar print generated in the four analyzed conditions.

According to Ferguson’s criteria [7], comparing the improvement of the Fixtoe Device® with and without metatarsal pad with that of tape intervention showed a moderate to high and moderate effect size for both peak pressure (Cohen’s d 1.45 and 2.36, respectively) and pressure–time integral reduction (Cohen’s d 1.86 and 2.53, respectively). The comparison between the reduction observed in both Fixtoe Device® modalities showed a weak effect size (Table 3).

**Table 3:** Interventions’ effect comparison

	**Pmax (Kpa)**				**P–T (Kpa/s)**			
	∆	Cohen’s d	95% CI	*p*-value	∆	Cohen’s d	95% CI	*p*-value
**Stabilizing Tape vs. Fixtoe Device®**	- 41.2 (± 6.5)	1.45	0.81 – 2.08	< .0001	- 8.8 (± 1.2)	1.86	1.19 – 2.54	< .0001
**Stabilizing Tape vs. Fixtoe Device® w/metatarsal pad**	- 60.0 (± 6.9)	2.36	1.62 – 3.1	< .0001	- 11.9 (± 1.3)	2.53	1.77 – 3.28	< .0001
**Fixtoe Device® vs. Fixtoe Device® w/metatarsal pad**	- 18.9 (± 5.3)	0.82	0.23 – 1.41	.005	- 3.1 (± 0.9)	0.68	0.1 – 1.26	.009

*Pmax* Maximal pressure, ∆ mean difference, *95% CI* 95% Cohen’s d confidence interval, *P/T* pressure–time integral. Mean (± SEM)

## Discussion

All interventions evaluated in this study were effective at reducing both maximal plantar pressure and pressure–time integral under the second metatarsal head in healthy individuals. However, the effect of the Fixtoe Device® interventions was higher than with the traditional second metatarsophalangeal joint stabilizing taping technique described by Yu et al [35].

The greatest ∆ was observed in the Fixtoe Device® with metatarsal pad intervention. The lowest ∆ was observed when placing the stabilizing tape. However, the most important variation in ∆ among the interventions was observed when placing the Fixtoe Device® without a metatarsal pad relative to the tape intervention. This intervention showed a strong size effect. When adding the metatarsal pad to Fixtoe Device® a low variation in ∆ was seen. The size effect of this intervention was moderate. Since we are the first to evaluate the effectiveness of this novel stabilization device, we cannot compare our data to previous studies.

The results we obtained with the Fixtoe Device® are similar to those reported by other authors when placing horseshoe discharges and metatarsal domes under central (second and third) metatarsal heads in a healthy population [8]. Reduction of peak pressure and the pressure–time integral values were obtained in both situations. Horseshoe discharges and metatarsal domes also showed positive results when investigating pain relief [1, 8, 13, 15, 19, 31]. The research by Poon and Love showed custom-made orthosis with metatarsal dome reduced plantar pressure under central metatarsal heads by up to 13% in metatarsalgia patients [31].

Nordisen et al. also reported a significant peak pressure decrease when placing a metatarsal dome (8.4% reduction) under the first metatarsophalangeal joint in asymptomatic *pes planus*patients, which was the most effective in comparison with other discharge pads [29]. Guldemond et al. found the effectiveness of the metatarsal dome in the reduction of peak pressure was higher when combined with a higher arch slope on customized insoles (18% versus 39% compared to the plain insole condition) [10].

Even though we did not evaluate it, we understand the placement of Fixtoe Device® might not have a relevant influence on the obtained data. While the effectiveness of other devices, such as discharges or metatarsal domes, depends on the precision of their placement in relation to the metatarsophalangeal joints, as Landorf et al. and Martínez-Santos et al. recently pointed out [22, 27]. Hastings et al. found that maximal peak pressure reduction (32 ± 16%) was achieved when the metatarsal dome was placed 6.1 to 10.1 mm proximal to the plantar aspect of the metatarsal head [13]. This location is highly variable. Therefore, we recommend that the placement of metatarsal domes is assessed individually [2, 10, 12, 22, 26, 27].

Our results show that the combination of both components of Fixtoe Device® was the intervention that generated the lowest values in the peak of pressure and pressure–time integral. Previous studies have shown a decrease in the forefoot’s load when placing cushioning materials (e.g., different Poron® and Plastazote combinations and foams) under the metatarsal heads [5, 12, 17]. Not only during normal gait, but also when running, metatarsal cushioning pads have been shown to produce a peak plantar pressure decrease in the forefoot, as Hähni et al. reported using instrumented insoles on their investigation in healthy recreational runners [12]. Our work supports that the placement of cushioning materials underneath the metatarsal heads – the cushioning metatarsal pad included in Fixtoe Device®- generates a larger reduction in the studied values in that area.

Nevertheless, we did not investigate the isolated effect of cushioning materials. Domínguez et al. found that the placement of different isolated absorbing energy materials (Pedilastik®, Poron Medical®, or Jogtene®) did not decrease mean pressure in the forefoot area or under the metatarsal heads, which they associated with the need to combine them with a discharge fenestration [5]. Given our results, the combination of both effects – cushioning and discharge – generated by Fixtoe Device® is more effective at reducing the load in metatarsal heads.

In clinical practice, cushioning materials, discharges, and metatarsal domes are not usually placed as an isolated element inside the patients’ shoes or on the foot but are included as elements of a complete foot orthosis. In Spain, the approximate cost of the novel device for the patient is around 45€, while the direct costs for a pair of insoles are usually higher than 126€.

Furthermore, we analyzed the effect of the interventions on the pressure–time integral. The relevance of the pressure–time integral measures is well known, and the duration of the load at a specific point might be more relevant than the magnitude of the pressure itself. Therefore, in the plantar aspect of the foot, the continuous application of a mild pressure trough time would be more significant than the brief application of higher pressures in pain occurrence [28]. The possible relationship between pressure–time integral and deformity progression likely supports the clinical relevance of our findings in propulsive metatarsalgia patients, and further investigations should address this.

Our results show that the placement of a stabilization tape according to the traditional technique on the second metatarsophalangeal joint reduced maximal plantar pressure and pressure–time integral in the second metatarsal head relative to the barefoot condition. This could be an explanation for the clinical improvement seen with this treatment by other authors [3, 18, 30, 35]. Nevertheless, we did not find any other studies quantifying the effects of the stabilization tape in terms of maximal pressure or pressure–time integral. In general terms, we also believe the elasticity and movable anchoring in the novel device offers the patient an easier fitting than the traditional tape.

This study has some limitations. In the first place, even though the results of epidemiological studies are not homogeneous, metatarsal pain seems to be more frequent in middle-aged women [6, 9, 11, 33]. Regarding our demographics, participants are younger, and gender distribution is nearly 50%. Due to the characteristics of the sample, the results of this study might not be transferable to real patients.

The pressure improvement achieved with Fixtoe Device® seems to be associated with its greater thickness. Nevertheless, future studies could also evaluate plantar pressure variation after a possible thickness reduction secondary to long-term use.

As a final reflection, we chose to carry out the investigation with healthy participants due to the availability of the sample, and since we believed changes should be first seen in individuals without deformity or pain. Due to the characteristics of the participants, even though we found good results with regard to plantar pressure, future work should evaluate the FixToe Device®’s effectiveness in patients with this alteration. Furthermore, our study did not analyze certain characteristics of the novel device that might have an influence on its effectiveness, such as the possibility that its size, particularly when used with the metatarsal pad, affects its correct placement, and the possible need for a larger space inside the patients’ shoewear. As such, future studies should verify the efficacy of Fixtoe Device® in propulsive metatarsalgia patients, including subjective measures, such as comfort with the novel device, pain, or inflammation relief as a result of measures in relationship with a decrease in maximal plantar pressure, as other authors did before with metatarsal pads [19].

To our knowledge, this is the first investigation proving the effectiveness of the recently developed Fixtoe Device® in terms of plantar pressure modification, which leads the way to its use in clinics.

## Conclusions

The Fixtoe Device® reduces median maximal pressure and median pressure–time integral under the second metatarsal head in healthy young individuals. The Fixtoe Device® shows higher effectiveness than the traditional second metatarsophalangeal joint stabilizing taping technique.

## Data Availability

The datasets used and/or analyzed during the current study are available from the corresponding author on reasonable request.

## Abbreviations

MTP: Metatarsophalangeal
MTPJ: Metatarsophalangeal joint
IQR: Interquartile range
BMI: Body mass index
FHL test: Functional *hallux limitus* test
STJ: Subtalar joint
Pmax: Maximal pressure
95% CI: 95% Confidence interval
P/T: Pressure-time integral
